# Personal Values Associated with Prosocial Decisions

**DOI:** 10.3390/bs10040077

**Published:** 2020-04-15

**Authors:** Renata M. Heilman, Petko Kusev

**Affiliations:** 1 Department of Psychology, Babes-Bolyai University, 400015 Cluj-Napoca, Romania; 2 Behavioural Research Centre, University of Huddersfield, Huddersfield HD1 3DH, UK; p.kusev@hud.ac.uk

**Keywords:** prosocial decisions, inequality aversion, personal values, self-transcendence

## Abstract

Social situations require people to make complex decisions, sometimes involving different outcomes for the self and others. Considering the long-lasting interest scholars are showing in the topic of social decisions, the aim of the current article is to add to this research line by looking at personal values as possible factors associated with a preference for more self-maximizing or cooperative choices. In a general adult sample (*N* = 63), we used the Social Value Orientation (SVO) slider measure to investigate participants’ tendency towards prosocial or proself outcomes. We also administered a personal values questionnaire, measuring 19 basic values, organized in 4 higher-order values. Building on the theory of basic individual values, we expected self-transcendence to be positively associated with more prosocial orientations. Our main result confirmed that self-transcendence was positively correlated with SVO whereas no other higher-order values were associated with SVO. Our data also revealed that inequality aversion was the primary motivation of prosocials, and this result was unrelated to gender effects or the personal values under investigation.

## 1. Introduction

Social situations require people to make complex decisions, sometimes involving different outcomes for the self and others. Traditional economic theories, such as expected utility theory or game theory [1], assumed that people would be primarily interested in self-maximizing choices. Nevertheless, numerous research contributions from both psychology and economics refuted the self-maximization principle, indicating that people prefer quite frequently options that illustrate a more cooperative or fairness-inclined nature [2]. Some of the most investigated factors that divert decision-makers from following the self-maximization norm include decision context, content, experience, and emotions [3,4,5,6]. Considering the long-lasting interest scholars are showing in the topic of social decisions, the aim of the current article is to add to this research line by looking at personal values as possible factors associated with a preference for more self-maximizing or cooperative choices. Largely overlooked is the question of whether there is an association between personal values and prosocial decisions. Accordingly, the aim of the study was to investigate personal values as possible correlates of social value orientation, using two instruments (Portrait Value Questionnaire [7] and the Social Value Orientation slider measure [8]) with proven reliability and validity. This specific question has not yet been investigated in previous studies; our study aims to provide evidence that personal values can be considered relevant correlates of prosocial decisions.

Evolutionary theories suggest that cooperation between people might be an adaptive mechanism for the survival of the species, since throughout phylogenesis, numerous situations can be identified in which objectives are achieved more efficiently if people cooperate [9,10]. However, successful cooperation requires complicated decisions on how resources should be divided among collaborators. For this purpose, fairness norms are particularly important [6]. Judgements of fairness and intentions behind money allocation decisions are frequently invoked [11]. There is converging behavioral and neuroimaging data that indicates that people engage in fairness judgements [12] due to a concern for reciprocity [13] or inequity aversion [14,15]. Therefore, studies suggest people might have an innate sense of fairness that guides their behavior in social interactions and division of a benefit.

Different methodologies have been used to investigate cooperative behaviors in humans; this resulted in specific classifications of the decision agents faced with a social dilemma. The research tradition in social psychology on social value orientation (SVO) distinguishes between competitors, who are motivated to achieve better payoff than others; prosocials, who make choices that increase group welfare; and individualists, whose behavior aims at self-maximization [16]. SVO can be defined as stable preferences for certain pattern of outcomes for oneself and the others [16,17,18]. Studies have shown that the three SVO types predict behavior in various social dilemma tasks, with prosocials displaying more cooperation type behaviors while individualists and competitors show a preference to maximize their own gain or their gain relative to the other. [16,19]. More recent work [8,20] adds altruism as a distinct social dilemma behavioral type, indicating a preference towards maximizing the payoff to the other. Another important theoretical and methodological development regarding SVO types is related to the possibility to distinguish between two different prosocial motivations: joint maximization vs inequality aversion [8,20]. It is thought that a prosocial decision-maker is motivated by joint maximization when their decisions maximized joint payoffs whereas inequality aversion is inferred from decisions that maximize equality of outcomes for self and the other.

In the economic literature, we can also find significant decision-making tasks that can be used to classify people in terms of their self vs other-regarding preferences. For instance, using an experimental economics decision-making task of resource allocation between a private and a group account, Kurzban and Houser classified participants in three main categories: free-riders (who contribute 0 to the group account), cooperators (who contribute 100% to the group account), and reciprocators (whose contribution to the group account is at the same level as the average contribution of the other group members) [10,21]. A meta-analysis looked at the relationship between SVO and cooperation in social dilemmas revealing a small to moderate relationship between SVO and cooperation and highlights the importance of SVO as antecedent of cooperation. Irrespective of the experimental paradigm scholars adhere to, there is a consistency regarding a general distinction between a proself and prosocial decision-making preference in social dilemmas [22].

Although previous research has helped in clarifying possible underlying mechanisms of SVO and cooperative behavior (see, for instance, References [16,23]), there are still many questions left regarding what contributes to cooperation and if it can be linked to stable individual differences [10,16,23,24].

Personal values can be considered guidelines by which we evaluate different courses of action and outcomes [16,25]. The theory of basic human values [26] identifies a comprehensive set of human values that can be distinguished in any society. In several highly cited manuscripts, Schwartz and coauthors developed the Portrait Values Questionnaire (PVQ) [7,27] that classifies 10 and, in a more recent and fine-tuned version, 19 basic individual values as “trans-situational goals, varying in importance, that serve as guiding principles in the life of a person or group” [7] (p. 664). The 19 basic individual values are organized in a “motivational continuum” and grouped in four higher-order values based on the compatibility and similarity of the type of motivational goal they express. *Openness to change* values that emphasize readiness for new ideas, actions, and experiences are contrasted with *conservation* values that emphasize self-restriction, order, and avoiding change. *Self-enhancement* values that emphasize pursuing one’s own interests are diametrically opposed to *self-transcendence* values that emphasize transcending one’s own interests for the sake of others [7]. Previous research has linked the 10 initial or the more recently 19 basic individual values with specific everyday behaviors that the values supposedly motivated [28,29]. Although personal values are a research topic for an increasing number of studies, there are still relatively few articles investigating whether and when personal values influence behavior and, particularly, prosocial behaviors. In a review article [25], personal values conceptualized according to Schwartz’s value theory [7] were analyzed as predictors of prosocial behavior in various types of economic exchange tasks. Individual values included in the higher-order value of self-transcendence were positively related to prosocial behaviors in some of the investigated exchange tasks. The results of the studies included in the review provide evidence that Schwartz’s personal values are predictive of prosocial behaviors [25].

Without any doubt, many factors influence everyday behavior. However, research indicates that personal values constitute a significant source of influence, and new research is needed to clarify the specific conditions under which personal values predict real-life behaviors and what research instruments are appropriate to address this gap. Previous research has already indicated that Schwartz’s personal values predict prosocial behavior, as measured by decision-making tasks derived from the economic literature [25]. Additionally, the social psychology-inspired SVO tradition has also been under investigation as an antecedent of prosocial behavior [22]. We consider that it would be important to link these two lines of research by changing the lenses and by looking at prosocial behaviors inferred from SVO as a correlate of personal values. Our current study contributes to this topic by showing that self-transcendence values are positively associated with prosocial decisions as measured by an SVO task.

## 2. Materials and Methods

### 2.1. Participants

Sixty-three healthy adults from the general population voluntarily participated in the study (*N* = 26 female). The mean age was 31.92 (SD = 1.01). All participants signed an informed consent prior to their participation, and anonymity was guaranteed to all participants. The study protocol follows the guidelines of the Romanian Psychologist’s Code of Conduct [30].

### 2.2. Materials and Procedure

In the present study, we set out to investigate the relationship between personal values, especially self-transcendence, and the SVO as measured by the allocation of resources in a hypothetical decision-making task. To this purpose, we used the Portrait Value Questionnaire [7] and the Social Value Orientation slider measure [8].

Participants completed the SVO slider measure and filled in the PVQ scales online, using Qualtrics Survey Platform. Age and gender details were collected first, followed by the PVQ scales, and finishing with the 15 SVO slider measure items.

The SVO slider measure [8,20,31] contains six primary and nine secondary items. In each item, the participant is required to choose among 9 pairs of payoffs for the self and another participant. The six primary items allow for the assessment of a person’s general SVO on a continuous scale, in terms of an angle. Conveniently, the angles can be converted to the four traditional archetypes described in the literature: altruist, prosocial, individualist, and competitor. Larger positive angles indicate an increased positive concern about the payoff of the other person (i.e., increased prosociality), whereas negative angles correspond to a negative concern about the others’ outcome (i.e., increased competitiveness). Based on the coding procedure described by Murphy, Ackermann, and Handgraaf [8], the four archetypes would correspond to the following angles:

- *altruists* would have an angle larger than 57.15°,
- *prosocials* would have an angle between 22.45° and 57.15°,
- *individualists* would have an angle between −12.04° and 22.45°, and
- *competitors* would have an angle lower than −12.04°.

The set of nine secondary items distinguishes between tendencies of inequality aversion and joint outcome maximization among prosocials. The coding procedure of the secondary items yields an inequality aversion index (IA) raging between 0 and 1. A larger IA index represents an increased preference for maximum joint outcome, whereas an IA index of 0 corresponds to allocation choices perfectly consisted with inequality aversion. A detailed description of the SVO slider measure items and coding procedure can be found in Reference [8,20,31].

The revised Portrait Values Questionnaire (PVQ-RR) [7] consists of 57 gender-matched portraits of different people, each describing a goal that is important to that person. For each portrait, the participant is asked to indicate how similar the described person is to them on a 6-point Likert scale (1 = not like me at all; 6 = very much like me). The 57 portraits account for 19 distinct values, which can be subsumed to 4 higher order values. The 19 distinct values and the higher-order values accompanied by their conceptual definitions are listed in Table 1.

The PVQ-RR instrument has been tested in 15 samples from 10 different countries and the confirmatory factor and multidimensional scaling analyses supported the discrimination of the 19 values [7]. In addition, a validation study on Russian population confirmed the discrimination of the 19 values and correlated each of the values with distinct behaviors [28]. For the purpose of our study, the instrument has been translated in Romanian and independently back-translated to English.

## 3. Results

The participants were adult volunteers. In Table 2, we present the occupation distribution as reported by the participants in the study.

Table 3 summarizes the descriptive statistics including means and standard deviations for age, the PVQ scales, and SVO angle.

Independent sample t-tests for the PVQ scales and SVO angle revealed no significant gender effect, except for the Conservation scale, where female participants had higher scores than male participants t(61) = −2.3, *p* < 0.05 (M_female_ = 4.61, SD_female_ = 0.65; M_male_ = 4.23, SD_male_ = 0.64).

The average SVO angle as an indicator of prosociality, as generated from the six primary items of the SVO slide measure, was 29.88 (SD = 1.43, min = −0.82, max = 53.89). According to Murphy and collaborators [18], 46 participants with SVO angles between 22.45° and 57.15° can be classified as prosocial (*N* = 21 female) whereas the remaining 17 participants with SVO angles between −12.04° and 22.45° can be classified as individualists (*N* = 5 female). No altruist or competitive types were identified among our study sample (with SVO angles above 57.15° and below −12.04° respectively).

To investigate our hypothesis regarding the associations between SVO angles and personal values, we conducted a correlation analysis. The correlation matrix is presented in Table 4.

Confirming our hypothesis, self-transcendence has a positive correlation with SVO angle r(63) = 0.263, *p* < 0.05. This means that participants with increased self-transcendence also have an inclination towards more prosocial behaviors as measured by the resources sharing task. Although other correlations emerged between the constructs assessed by the PVQ scales, they were not significantly related to prosocial behavior.

**Figure 1:**
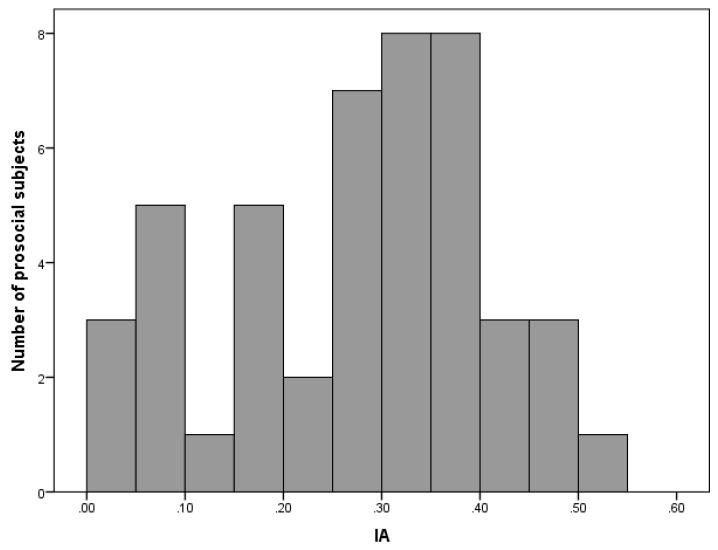
Number of participants displaying prosocial preferences from inequality aversion (0) to joint gain maximization (1).

The nine secondary items of the SVO slier measure were explicitly designed to disentangle the prosocial motivations of joint maximization from inequality aversion [18]. Our sample included 46 participants categorized as prosocials. Among this subsample, an inequality aversion (IA) index was computed, as indicated by Reference [8]. The IA index ranges between 0 and 1, with lower values indicating allocation choices consistent with inequality aversion and higher values indicating allocation choices consistent with a preference for joint maximization. Within our sample, IA index had a mean of 0.266 and SD = 0.02 (min = 0.00; max = 0.53), indicating that participants ranged more towards the inequality aversion motivation underlying prosocial distributions. No IA-significant gender effects emerged (M_female_ = 0.26, SD_female_ = 0.15; M_male_ = 0.26, SD_male_ = 0.13). The participants’ distribution regarding the IA index is illustrated in Figure 1. None of the PVQ scales were significantly correlated with IA.

## 4. Discussion

Social preferences have a major impact on multiple everyday activities. In this study, we aimed at investigating the association between personal values and SVO in an adult sample from the general population. The personal values under consideration were described by Schwartz [26] in his formulation of the theory of basic individual values, and they have been largely studied in relation to everyday behaviors. To account for the typical prosocial vs proself orientations in social dilemmas, we used the SVO slider measure [8]. Building on the theory of basic individual values, we expected self-transcendence to be positively associated with more prosocial orientations.

The theory of basic human values [26] identifies a set of 19 individual values that are distinguishable in terms of their motivational goals. The individual values have been linked to specific everyday behaviors [7,25,28]. Personal values assessed with the PVQ [7] have also proven their significant effect in facilitating behavior in applicative settings, such as participating in peer-to-peer platforms for sharing and circulating resources [32,33]. Building on previous research successfully using the PVQ as an instrument to analyze personal values as antecedents for human behavior, we also opted for using this scale. Our main result confirmed that self-transcendence was positively correlated with SVO, whereas no other higher-order values were associated with SVO. Our study is in line with previous work, indicating that personal values are systematically related to everyday behaviors that are distinguishable in terms of their motivational goals [7,25,28].

Murphy’s [8] method to classify participants according to their SVO angle generates four behavioral types differing in their preferences towards prosocial vs proself payoff allocations, namely altruist, prosocial, individualist, and competitive. Previous research using the SVO slider measure has shown that prosocial and individualist types are relatively more frequently encountered than the more altruist and competitive types [8,20,34]. However, in our study sample, only prosocial and individualist types were encountered. Aside from evaluating personal values, only age and gender information were collected from participants, and they were unrelated to SVO. Therefore, it is possible that some unassessed cultural or individual differences could be related to the specific SVO types composition we found in our sample.

Within the prosocial subsample, the SVO slider measure permits the distinction between two different underlying motivations, namely inequality aversion and joint maximization. Our data revealed that inequality aversion was the primary motivation of prosocials, and this result was unrelated to gender effects or the personal values under investigation. Our result corroborates the idea that people value equality and fairness [6,35]. Based on our existing data, we cannot speculate what relevant inequality aversion antecedents could have been. Future studies, with a more complex design and an increased list of individual differences measurements could continue this line of research.

One noteworthy aspect of the current study is that we investigated a sample from the adult general population not a convenient student sample. Student samples consisting of same age and college major participants can be expected to be homogenous in terms of personality traits and personal values [24,36]. We can speculate this is not the case within our sample, since we included general population adults, with different career choices and age. The aim of the study was to investigate personal values as possible correlates of social value orientation, using two instruments that have proven their relevance to the scientific community. Accordingly, this specific relationship has not yet been investigated in previous studies.

An important limitation of our study resides in its correlational nature, thus not allowing to formulate any cause-and-effect relation between SVO and personal values. Nevertheless, studies have shown that individual SVO, as measured by the SVO slider measure, changes in response to external factors, such as information about the intentions or past behavior of the person who would be affected by the decision-making outcomes of the participants [20]. Therefore, we could speculate that personal values, along with other individual differences, could be predictors of SVO but that future studies are needed to further investigate this topic. Numerous previous studies have provided ample support for the influence of personality traits, current affective state, and dispositional mood on decision-making [5,6,9,21,23,37,38]. Our aim for the present study was to complement the existing literature on these highly investigated individual differences in decision-making with the less investigated line of research focusing on personal values, thus proving new evidence that human decisions are influenced by a large category of internal and external factors.

In conclusion, our study contributes to the developing literature looking at the factors associated to cooperation in social dilemmas by analyzing personal values as possible correlates. Supporting the theory of basic individual values [7], our results show that the higher-order value of self-transcendence is a significant positive correlate of prosocial behaviors in a resources allocation task.

## Figures and Tables

**Table 1:** The 19 values and 4 higher order values and their conceptual definitions adapted from Reference [7].

Higher Order Value	Value	Conceptual Definitions in Terms of Motivational Goals
Openness to change	Self-direction–thought	Freedom to cultivate one’s own ideas and abilities
	Self-direction–action	Freedom to determine one’s own actions
	Stimulation	Excitement, novelty, and change
	Hedonism	Pleasure and sensuous gratification
Self-Enhancement	Achievement	Success according to social standards
	Power–dominance	Power through exercising control over people
	Power–resources	Power through control of material and social resources
Conservation	Face	Security and power through maintaining one’s public image and avoiding humiliation
	Security–personal	Safety in one’s immediate environment
	Security–societal	Safety and stability in the wider society
	Tradition	Maintaining and preserving cultural, family, or religious traditions
	Conformity–rules	Compliance with rules, laws, and formal obligations
	Conformity–interpersonal	Avoidance of upsetting or harming other people
	Humility	Recognizing one’s insignificance in the larger scheme of things
Self-Transcendence	Benevolence–dependability	Being a reliable and trustworthy member of the ingroup
	Benevolence–caring	Devotion to the welfare of ingroup members
	Universalism–concern	Commitment to equality, justice, and protection for all people
	Universalism–nature	Preservation of the natural environment
	Universalism–tolerance	Acceptance and understanding of those who are different from oneself

**Table 2:** Participants’ occupation distribution according to the International Standard Classification of Occupations (ISCO-08).

ISCO-08 Job Category		Count
**1**	Managers	0
**2**	Professionals	28
**3**	Technicians and associate professionals	6
**4**	Clerical support workers	7
**5**	Service and sales workers	6
**6**	Skilled agricultural, forestry and fishery workers	0
**7**	Craft and related trades workers	7
**8**	Plant and machine operators and assemblers	4
**9**	Elementary occupations	0
**10**	Armed forces occupations	2
	Unemployed	3

**Table 3:** Descriptive statistics.

Variable	Overall		Male		Female	
	Mean	SD	Mean	SD	Mean	SD
Age	31.92	1.01	32.24	7.59	31.46	8.81
Self-Transcendence	4.83	0.08	4.71	0.69	5.01	0.65
Self-Enhancement	4.13	0.1	4.04	0.86	4.26	0.81
Openness to Change	4.79	0.08	4.67	0.62	4.97	0.66
Conservation	4.39	0.08	4.23	0.64	4.61	0.65
SVO angle	29.88	1.43	28.35	12.62	32.05	9.23

Note: SVO angle = social value orientation angle.

**Table 4:** Correlation matrix between SVO angles and Portrait Values Questionnaire (PVQ) scores.

Variable	1	2	3	4	5
1. SVO angle	-				
2. Self-Transcendence	0.263 *	-			
3. Self-Enhancement	−0.186	0.066	-		
4. Openness to Change	−0.030	0.489 **	0.371 **	-	
5. Conservation	0.780	0.609 **	0.500 **	0.265 *	

Note: * Pearson correlation is significant at *p <* 0.05. ** Pearson correlation is significant at *p <* 0.01.
